# Psychotropic consumption before and during COVID-19 in Asturias, Spain

**DOI:** 10.1186/s12889-023-15360-0

**Published:** 2023-03-15

**Authors:** María Luisa Nicieza García, Paula Fernández Martínez, Eva Fernández Bretón, Marta M. Martínez Alfonso, Patricio Suárez Gil

**Affiliations:** 1 Health Department, General Directorate of Health Policy and Planning, Asturias, Spain; 2 Biostatistics and Epidemiology Platform, Health Research Institute of Asturias, Asturias, Spain

**Keywords:** Primary health care, Anti-anxiety agents, Antidepressive agents, Hypnotics and sedatives, Drug prescriptions

## Abstract

**Background:**

Spain as multiple other countries has been experiencing an increasing and sustained trend in the use of psychotropic medications since the mid 90s. Recent studies show public health measures implemented to control SARS-Cov2, such as mobility restrictions and the shutdown of nonessential activities increased mental suffering, even contributing to a higher number of anxiety, depression and insomnia disorders that could lead to an increase in the consumption of psychotropics.

The aims were: 1) Evaluate the temporal trend in psychotropic consumption by pharmacological subgroup, sex, and age group 2) Estimate the effect of the COVID-19 pandemic in the use of psychotropic drugs.

**Methods:**

We conducted a retrospective observational study, retrieving all prescriptions of anxiolytics, hypnotics and sedatives, and antidepressants dispensed in pharmacies of Asturias (Northern Spain) for Primary Care patients for the period 2018–2021.

We presented the data expressed in Daily Defined Doses (DDDs) for 1000 persons/day (DHD). To estimate changes in DHDs by year and age group we conducted two multiple linear regressions (one for males and one for females) for every pharmacological subgroup studied. Changes were considered statistically significant when the regression coefficient was *p* < 0.05. We used the Software R 4.1.0.

**Results:**

For the studied period, the highest DHDs are for antidepressants, although all of the subgroups experienced an increase in consumption rates.

Women consumed more psychotropic drugs than men. In 2021, 372 out of every 1000 women were taking daily 1 DDD of these drugs versus 184 out of every 1000 men.

Consumption rates for all psychotropic drugs progressively increases with age. Conversely, the biggest increases in consumption were among the youngest age groups (0–14 and 15–29 years) for women, while for men there is more variability.

The regression models suggest an upward trend in psychotropic consumption during all the period, especially remarkable from 2020, for both genders and all age groups.

**Conclusions:**

- The consumption of psychotropic drugs has gradually increased over the last 4 years, with a significant boost starting in 2020 for both sexes, matching the start of the SARS-COV2 pandemic and the implementation of strict Public Health measures to contain it.

- The increase observed on children and adolescents is a matter of concern.

## Introduction

Spain, as many other countries, has been experiencing an increasing and sustained trend in the use of psychotropic medications since the mid 90s. This trend has been a matter of concern and constant reviews for regulatory agencies and healthcare providers worldwide [1–4].

A survey conducted in Spain by the National Observatory for Drugs and Addictions (OEDA) estimates that prevalence for consumption of prescription and over-the-counter hypnotics and sedatives at some point in life was 18.7, 20.8 and 22.5% in 2015, 2017 and 2019 respectively [5].

The latest report of the International Narcotics Control Board showed in 2020 that Spain, followed by Belgium and Portugal, led the world in licit consumption of anxiolytics and hypnotics and sedatives [6]. According to the latest official data from the Ministry of Health, in Spain the consumption of anxiolytics and hypnotics and sedatives, increased by 5.9 and 6.6% respectively in 2021 compared to 2018 [7]. For the case of antidepressants the official data published by the Ministry of Health for Spain, are from 2013 [2].

Additionally, some studies reveal a clear female-to-male ratio in psychotropic consumption, with women consuming twice as much as men. The report [8] “Mental Health in data” pointed out that 34% of women older than 40, versus 18% of men of the same age range were prescribed at least a psychotropic drug in 2017. The same report shows that prevalence rates for anxiety, depression and insomnia were higher in women.

Secades Villa R y cols [9], estimate that 37.9% of 602 patients surveyed in Primary Care in Asturias from October to December 2002 were taking psychotropic drugs or consumed them in the previous month. Among those used these medications in the last year, 60% were taking anxiolytics or hypnotics and sedatives exclusively, 9% were taking antidepressants only and 31% were taking both.

A Spanish study from Henares Montiel et al. [10]. (2020) points out an important variability on psychotropic prescriptions among Spanish regions, additionally noting that women scored worse for every mental health indicator that was measured for the study, such as diagnosis of mental disorder, self-perceived health, and psychiatric morbidity. Moreover, the study presents rates of hypnotics and sedatives consumptions for women that were twice as those for men in almost every region, and even three times higher in 4 regions, Asturias being one of them.

Recent studies published in Spain [11, 12] and other countries [13, 14] point out that public health measures implemented to control SARS-Cov2, such as mobility restrictions school closures and the shutdown of nonessential activities increased mental suffering, even contributing to a higher number of diagnoses of anxiety, depression, insomnia, and post-traumatic stress syndrome that could lead to an increase in the consumption of psychotropics. This was particularly true in women and younger age groups. Some studies suggest that prevalence of these disorders has increased considerably in children and adolescents during the COVID-19 pandemic [15–18].

Asturias is one of the regions in Spain with the highest mental health burden, which is reflected in direct indicators such as the suicide rates (the highest in Spain) [19] but also in proxies like the consumption of psychotropic drugs, as mentioned earlier in this paper.

The impact of the COVID-19 pandemic on the mental health of populations differs according to many factors [20]. It is possible that populations with a high burden of mental health problems prior to the pandemic may be disproportionately affected by an event like that.

We consider it relevant to study the impact of the pandemic on the evolution of an indirect indicator of mental wellbeing, such as psychotropic dispensation, in a previously burdened community from a mental health perspective.

Other studies have explored changes on dispensation and consumption of psychotropic drugs after the pandemic in different population subgroups, but not many of them quantify these effects after taking into account the differences across sex and age groups. Moreover, very few of the studies published until the moment include data from populations younger than 18. Prescription of psychotropic medication in this specific demographic has been a matter of concern in recent years, and there is substantial evidence indicating that the pandemic had a particularly significant effect on the mental health of children and adolescents [18].

In this sense, an active surveillance of psychotropic prescriptions is key in order to know how these drugs are used.

The aims of this study were:

- Evaluate the temporal trend in psychotropic consumption by pharmacological subgroup, sex, and age group.
- Estimate the effect of the COVID-19 pandemic in the use of psychotropic drugs in Asturias.

## Methods

### Setting

The region of Asturias is located in the north of Spain. As of 2021, it has a population of 1,011,792 [21]. Historically, the main industries in this region were mining and metallurgy. Starting in the 70s, there has been a process of deindustrialization in Asturias, which has had an impact on the region’s economy [22]. Asturias is also facing challenges related to an aging population, with a higher proportion of older people compared to the rest of Spain. (27.03% of the population in Asturias are older than 65 versus the national average of 20,08% in 2022) [23]. This is mainly due, on the one hand, to the fact that the birth rate is among the lowest in the country, and on the other hand, to the fact that young people emigrate to regions where jobs are available [24].

### Study design

We conducted a retrospective observational study, retrieving all prescriptions of pharmacological subgroups N05B (Anxiolytics), N05C (hypnotics and sedatives), and N06A (antidepressants), in accordance with the Anatomical Therapeutic Chemical (ATC) of the World Health Organization (WHO) classification system [25] dispensed in pharmacies through official prescriptions from the Asturias Healthcare Service (SESPA), for the Primary Care setting.

The Pharmacy Department of the Subdirectorate of Infrastructures and Technical Services of SESPA data referring to Defined Daily Dose (DDDs) and number of boxes were retrieved for the period 2018–2021. We didn’t include data from specialized care prescriptions or from private healthcare providers.

We presented the data as the number of boxes dispensed and annual consumption rate, expressed in DDDs for 1000 persons/day (DHD), which provides an estimation of how many people in 1000 are receiving a DDD per day.

Standard DDD is the technical unity of measurement recommended by the World Health Organization (WHO) for drug utilization studies and is defined as the assumed average maintenance dose per day for a drug used for its main indication in adults and for a specific route of administration (24). We used the following formula to estimate DDDs:

$$\textrm{DDD}=\textrm{amount of active principle consumed}/ \textrm{Standard DDD provided by WHO}$$

Data were expressed as DDDs per 1000 inhabitants per day (DHD), using the following formula:

$$\textrm{DHD}=\textrm{DDD}\times 1000\ \textrm{inhabitants}/\textrm{total}\ \textrm{population}\times \textrm{time}\ \left(\textrm{days}\right).$$

To calculate the DHD, we used the population with an active Health Card, obtained through the Healthcare Resources and Population Information System (SIPRES) for each year of the study. As of January 1st 2021, the total number of people recorded by this system was 1,002,067.

A locally estimated scatterplot smoothing (loess) approach was performed to represent DHDs consumption for men and women during the study period.

To estimate changes in DHDs by year and age group we conducted 2 multiple linear regressions (one for males and one for females) for every pharmacological subgroup studied (antidepressants, anxiolytics, and hypnotics and sedatives). Changes were considered statistically significant when the regression coefficient was *p* < 0.05. We used the Software R 4.1.0 (The R Foundation, Viena, Austria) [26].

We compared changes in prescriptions before and after COVID-19 onset within age groups by calculating yearly increases. We considered the 2018–2019 period as the prepandemic era, and we compared this increase with the average of 2020–2019 and 2021–2020 increases as the pandemic period.

The study got the Principality of Asturias Ethics Committee (CEImPA) approval (code 2022.229). This Committee agreed to the exemption of informed consent, as we used anonymized aggregated data.

## Results

Over the period 2018–2021, an annual average of 3,911,281.5 boxes of anxiolytics, hypnotics and sedatives and antidepressants were dispensed, and the consumption rate was 282.2 DHDs in 2021.

As shown in Table 1, total psychotropic consumption went from 251.1 DHD in 2018 to 282.2 in 2021, increasing 12.4% in this time period.

**Table 1:** Evolution of the DHDs by pharmacological subgroups in the period 2018–2021

Pharmacological subgroup Name and code (ATC)	DHD 2018	DHD 2019	DHD 2020	DHD 2021	∆ DHD 2021/2018 (%)
**Anxiolytics** **N05B**	104.7	104.4	109.1	113.7	8.7
**Hypnotics and sedatives** **N05C**	31.8	32.6	34.6	36.6	15.3
**Antidepressants** **N06A**	114.6	118.8	123.9	131.9	15.1
**TOTAL**	**251.1**	**255.8**	**267.6**	**282.2**	**12.4**

Source: billing records of medical prescriptions from SESPA. Total population with Health Card as of January 1st for each year

In the study period, the highest DHDs are for antidepressants, although all of the subgroups experienced an increase in consumption rates.

**Fig. 1:**
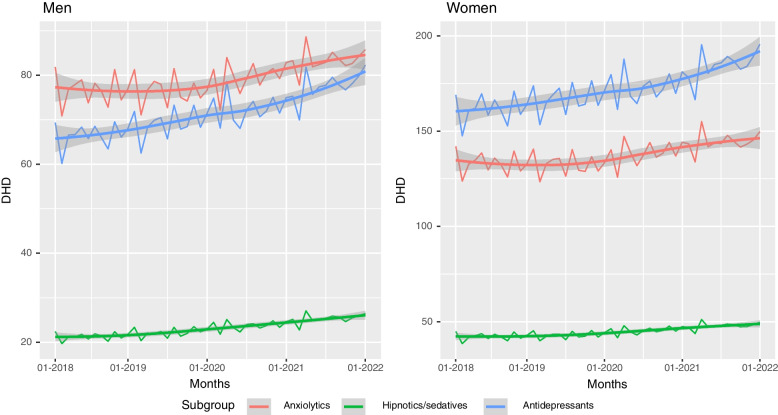
Evolution of DHDs by pharmacological subgroup and gender for the period 2018–2021

An upward trend on consumption was observed for the three subgroups of drugs studied and for both sexes, steeper for anxiolytics and antidepressants (Fig. 1). The kind of psychotropic most consumed for men was anxiolytics, with a DHD of 75.6 at the beginning of the study period and 82.2 at the end. The second most prescribed subgroup for men were antidepressants (65.6 at the beginning of the study period and 76.6 at the end, almost reaching the levels of anxiolytics consumption). However, for women, the most consumed psychotropics were antidepressants, which also showed an upward trend (159.5 versus 182.3), whereas anxiolytic consumption increased from 131.3 to 142.5. Hypnotics and sedatives show similar upward trends both for men and women, ranging from 21 to 25 and 41.7 to 47.2 respectively in the study period.

When analyzing the DHDs by sex and age (Table 2), except for the 0–14 age group, higher DHDs are found for women compared to men. Overall, for both men and women, the highest DHDs are found in the older age groups (75–89 and 90 or older). Only males in the 0–14 group experienced a decrease in the prescription of anxiolytics in the study period (− 14.3%).

**Table 2:** DHDs for pharmacological subgroups by age and sex in the period 2018–2021

***SUBGROUP*** ***ATC***	***AGE GROUP***	***DHD 2018*** ***SEX***		***DHD 2019*** ***SEX***		***DHD 2020*** ***SEX***		***DHD 2021*** ***SEX***		***∆ 2021/2018 (%)***	
		MEN	WOMEN	MEN	WOMEN	MEN	WOMEN	MEN	WOMEN	MEN	WOMEN
***Anxiolytics*** ***N05B***	0–14	0.7	0.6	0.7	0.8	0.6	0.7	0.6	0.8	−14.3	33.3
	15–29	9.7	12.0	10.5	12.2	11.2	14.1	12.34	16.3	27.2	35.8
	30–44	54.6	63.4	52.8	63.6	55.7	68.8	55.9	70.4	2.4	11.0
	45–59	120.1	155.4	118.9	152.0	120.9	157.1	123.5	159.1	2.8	2.34
	60–74	109.8	225.2	110.6	222.5	115.7	228.4	121.5	232.7	10.7	3.3
	75–89	116.1	235.9	114.6	234.2	117.6	241.8	124.7	253.0	7.4	7.2
	≥ 90	129.9	212.4	126.5	210.3	129.8	218.4	145.5	250.2	12.0	17.8
***Anxiolytics total age group***		**75,6**	**131,3**	**75,5**	**130,8**	**78,6**	**136,9**	**82,2**	**142,5**	**8,7**	**8,5**
***Hypnotics and sedatives*** ***N05C***	0–14	0.0	0.0	0.0	0.0	0.0	0.0	0.0	0.0	0.0	0.0
	15–29	1.8	2.2	2.0	2.2	2.1	2.7	1.9	3.2	5.6	45.5
	30–44	6.9	11.3	7.4	11.5	8.3	13.3	9.0	13.8	30.4	22.3
	45–59	21.6	35.0	22.2	35.9	24.0	38.4	24.8	40.3	14.8	15.3
	60–74	38.8	70.5	39.9	70.4	41.6	73.0	44.1	73.4	13.7	4.1
	75–89	63.6	109.1	64.9	109.7	66.8	111.6	69.2	116.2	8.8	6.5
	≥ 90	83.5	112.1	82.6	111.8	86.8	118.4	100.1	138.2	19.9	23.3
***Hypnotics and sedatives*** ***total age group***		**21,0**	**41,7**	**21,9**	**42,4**	**23,4**	**44,9**	**25,0**	**47,2**	**19,0**	**13,2**
***Antidepressants N06A***	0–14	0.6	0.6	0.6	0.7	0.7	0.7	0.8	1.2	33.3	100.0
	15–29	12.5	21.6	14.5	23.2	15.5	25.5	16.4	30.3	31.2	40.3
	30–44	43.4	77.8	44.4	80.5	46.3	84.1	47.3	87.8	9.0	12.9
	45–59	89.3	176.5	92.3	179.8	96.3	184.9	101.5	193.6	13.7	9.7
	60–74	98.0	260.3	101.2	264.9	105.2	270.7	113.5	281.3	15.8	8.1
	75–89	135.1	318.8	138.1	328.5	140.2	337.1	148.2	357.0	9.7	12.0
	≥ 90	157.5	278.6	165.8	295.5	171.4	306.1	192.5	352.4	22.2	26.5
***Antidepressants total age group***		**65,6**	**159,5**	**68,3**	**164,9**	**71,5**	**171,2**	**76,6**	**182,3**	**16,8**	**14,3**
***Total pharmacological Subgroup***		***162,3***	***332,4***	***165,7***	***338,2***	***174,0***	***353,0***	***184,0***	***372,0***	***13,4***	***11,9***

Globally, we didn’t find significantly different increases by sex for any of the pharmacological subgroups studied. The changes in prescription rates in the period 2019–2021 were comparable for men and women in the case of anxiolytics and antidepressants, although the rise for hypnotics and sedatives in women was greater (23% versus 14% in men).

By age, the largest increases in DHDs in the study period are found in children or young adults for women, while for men there is more variability: for anxiolytics, the increase is highest in the 15–29 age group (27%), for hypnotics and sedatives in mid-life (30.4% in 30–44 year olds), and again in the 15–29 age group for antidepressants (30%).

For the youngest age group, there was a 21% yearly rise after the pandemic onset versus 0% rise prepandemic for antidepressant prescription in males, and 35,5% versus 17% for females. These differences between periods were not the case for the other pharmacological subgroups studied. For young adults, we also observed a worrisome rise on the three pharmacological subgroups studied when compared with increases in the prepandemic period, but only for women (15% yearly increase in the period 2019–2021 versus 1,7% increase in the period 2018–2019 for anxiolytics, 19% versus 0% for hypnotics and sedatives, and 15% versus 7% for antidepressants).

As shown in Table 3, a positive correlation between DHDs and years was observed for all regression models. R2 values (which translates to the percentage of the variance explained by the model) were 0.99 or higher.

**Table 3:** Results of the multiple linear regression models for DHDs

			Men			Women		
			Estimate	CI 95%	P	Estimate	CI 95%	P
**Antidepressants**	Year	*2018*	Ref.			Ref.		
		*2019*	0.19	0.06–0.32	0.005	0.42	0.14–0.70	0.004
		*2020*	0.40	0.27–0.53	< 0.005	0.89	0.61–1.17	< 0.001
		*2021*	0.74	0.61–0.87	< 0.005	1.76	1.48–2.04	< 0.001
	Age	*0–14*	Ref.			Ref.		
		*15–29*	0.85	0.67–1.02	< 0.001	1.40	1.03–1.77	< 0.001
		*30–44*	4.64	2.95–6.33	< 0.001	8.47	8.10–8.84	< 0.001
		*45–59*	11.31	11.14–11.48	< 0.001	22.73	22.36–23.01	< 0.001
		*60–74*	10.04	9.87–10.21	< 0.001	29.65	29.28–30.02	< 0.001
		*75–89*	6.24	6.07–6.41	< 0.001	23.12	22.75–23.49	< 0.001
		*90 and older*	0.80	0.63–0.97	< 0.001	4.14	3.77–4.51	< 0.001
**Anxiolytics**	Year	*2018*	Ref.			Ref.		
		*2019*	−0.01	−0.13-0.11	0.88	−0.03	−0.23-0.16	0.77
		*2020*	0.20	0.08–0.32	0.001	0.44	0.24–0.63	< 0.001
		*2021*	0.44	0.32–0.56	< 0.001	0.88	0.68–1.08	< 0.001
	Age	*0–14*	Ref.			Ref.		
		*15–29*	0.62	0.46–0.78	< 0.001	0.75	0.49–1.01	< 0.001
		*30–44*	5.61	5.45–5.77	< 0.001	6.82	6.56–7.08	< 0.001
		*45–59*	14.42	14.26–14.58	< 0.001	19.29	19.03–19.55	< 0.001
		*60–74*	11.00	10.84–11.16	< 0.001	25.00	24.74–25.26	< 0.001
		*75–89*	5.25	5.09–5.41	< 0.001	16.62	16.36–16.88	< 0.001
		*90 and older*	0.61	0.45–0.78	< 0.001	2.98	2.72–3.24	< 0.001
**Hypnotics and sedatives**	Year	*2018*	Ref.			Ref.		
		*2019*	0.06	0.02–0.10	0.004	0.06	−0.01-0.13	0.10
		*2020*	0.17	0.13–0.21	< 0.001	0.25	0.18–0.32	< 0.001
		*2021*	0.28	0.24–0.32	< 0.001	0.44	0.37–0.51	< 0.001
	Age	*0–14*	Ref.	0.06–0.18		Ref.		
		*15–29*	0.12	0.76–0.88	< 0.001	0.14	0.04–0.24	< 0.001
		*30–44*	0.82	2.71–2.83	< 0.001	1.28	1.18–1.38	< 0.001
		*45–59*	2.77	3.91–4.03	< 0.001	4.63	4.53–4.73	< 0.001
		*60–74*	3.97	2.90–3.02	< 0.001	7.91	7.81–8.01	< 0.001
		*75–89*	2.96	0.37–0.49	< 0.001	7.70	7.60–7.80	< 0.001
		*90 and older*	0.43	0.06–0.32	< 0.001	1.62	1.52–1.72	< 0.001

Multiple linear regression models, adjusted for age and sex for each of the pharmacological subgroups, show that, in the case of anxiolytics and hypnotics/sedatives, there is only a statistically significant positive association between the years 2020 and 2021 and the consumption of DHDs, taking 2018 as a reference. In the case of antidepressants, there is a positive association with all years of the study, but the coefficients for 2020 and 2021 double each year.

Overall, these models suggest that although there has been an upward trend in psychotropic consumption from 2018 to 2021, this trend is especially noticeable from 2020 onwards, for both genders and all pharmacological subgroups.

## Discussion

According to the latest official data from the Ministry of Health [7], the increase in the consumption of anxiolytics in Spain in 2021 compared to 2018 was lower than that observed in our study in Asturias (5.9% vs. 8.7%, respectively), and the same is true for the consumption of hypnotics and sedatives (6.6% vs. 15.3%) as shown in Table 4. As previously stated, Asturias is one of the Spanish regions with the highest consumption of psychotropic drugs, and the fact that the increase in the study period is higher than the national average means that this trend is not only not reversing, but worsening.

**Table 4:** Consumption of psychotropic drugs (DHD) by pharmacological subgroup in Asturias and Spain

Subgroup ATC	Geographic area	DHD 2018	DHD 2019	DHD 2020	DHD 2021	∆ 2021/2018 (%)
**Anxiolytics**	Asturias	104,7	104,4	109,1	113,7	8,7
	**Spain**	**55,6**	**54,8**	**57,2**	**58,9**	**5,9**
**Hypnotics and sedatives**	Asturias	31,8	32,6	34,6	36,6	15,3
	**Spain**	**32,0**	**32,1**	**33,4**	**34,1**	**6,6**
**Antidepressants**	Asturias	114,6	118,8	123,6	132,0	15,1
	**Spain**	**No data**	**No data**	**No data**	**No data**	**No data**

Our models suggest that, similarly to national data, consumption of psychotropic drugs has gradually increased over the last 4 years, with a significant boost starting in 2020, matching the implementation of strict Public Health measures to contain the pandemic [27].

This is consistent with previous evidence pointing out a rise in the prescribing of psychotropic drugs at the national and international level [28–31]. However, some studies found an immediate drop in psychotropic drug prescription followed by a subsequent increase [32, 33], which could be attributed to a decreased number of doctor visits due to mobility restrictions. As we analyzed data on a yearly basis, we were not able to detect an immediate decline in prescribing, but we identified a positive time trend over the whole study period which accelerated during the first 2 years of the pandemic.

In our study, the pharmacological subgroup with the highest DHDs is antidepressants, followed by anxiolytics. Regarding antidepressant consumption, there are not national data available yet to establish a comparison (last official data was published in 2013). Nevertheless, a study from Andalusia [30], another Spanish region, showed an increase of 3.7% between March 2019–February 2020 and March 2020 to February 2021.

The use of psychotropic drugs among women is striking, being almost twice as high as among men in all three subgroups. Several studies remark that the gender gap on psychotropic drug prescription was accentuated during the pandemic. Estrela et al. found a rise just in the prescription of anxiolytics, hypnotics and sedatives among women older than 65 in Portugal [33].

A US cohort study of 100.000 patients found women had larger changes in prescription rates over time [34], and the authors conclude that sex disparities may have been exacerbated after the pandemic onset. In our study, the changes in prescription rates in the period 2019–2021 were comparable for men and women in the case of anxiolytics and antidepressants, although the rise for hypnotics and sedatives in women was nearly two times higher.

Several hypotheses that can explain the higher consumption rates for women can be found in the literature, ranging from more medical consultations [35–37], to gender inequalities regarding the double working shift, and their role of caregivers [38, 39], which would translate into more psychological stress and explain higher prevalence of anxiety, depression, and sleep and eating disorders in women [8].

Regarding age, consumption is higher for older age groups. These results are similar to those reported on a study by Gonzalez-Lopez [30], with data from another Spanish region, Andalusia.

Our results, as in the study by Gonzalez-Lopez et al. [30] show psychotropic consumption increases with age in both genders. A plausible explanation for this would be chronic disease and comorbidities that come with aging, alongside cognitive decline. All these factors could lead to more depression, anxiety, isolation and physical dependency. These, combined with a greater use of healthcare services could explain a higher use of psychotropic medications.

Some studies point out that there has been a concerning increase in psychotropic consumption in younger age groups during the pandemic [40]. In our study, when comparing increases in the pandemic period versus the prepandemic period, we found that this was the case for antidepressants, but not for the other subgroups studied. For young adults, we also observed a worrisome rise on the three pharmacological subgroups studied, but only for women.

To this regard, several studies suggest a surge in the prevalence of mental health disorders in childhood and adolescence during COVID-19 [15–18], with depression, anxiety, sleep and eating disorders being the most common. Although this phenomenon can be partially due to previous vulnerability factors, linked to the development of less resilient personality structures, the pandemic has had an important impact on stress levels [18], and, compared to adults, this impact can last longer, which could explain the growing trend of psychotropic consumption in this particular demographic. Given the potential long term effects of these medications, including addiction in the case of benzodiazepines, this should be of great concern.

One of the limitations of our study was that we were unable to analyze the prescriptions made by private healthcare providers, which could not be quantified because the information was not available and was not part of the objectives of the study, so that the real consumption will nevertheless be higher than that obtained.

In addition, it is worth mentioning that, for our study, we gathered data of the drugs dispensed, once they have been prescribed by the Primary Care Physicians. The exact use of these drugs is very difficult to assess. Additionally, to over-the-counter consumption, adherence must be taken into account, which modulates the real or effective consumption. However, we consider the information provided by dispensation as a good proxy of actual consumption, given that the sale of this type of drugs over-the-counter is heavily regulated and most of the prescriptions come from Primary Care.

## In conclusion

- The consumption of psychotropic drugs has gradually increased over the last 4 years, with a significant boost starting in 2020 for both sexes, matching the start of the SARS-COV2 pandemic and the implementation of strict Public Health measures to contain it.
- The increase in the use of psychotropic drugs in children and adolescents during the pandemic is a cause for concern, given the addictive power and long term effects of these drugs.
- The evaluation and publication of data on the consumption of psychotropic drugs is of great relevance for health policy makers and essential for the information of professionals and the general public, to guide areas for improvement and establish the appropriate corrective measures.

## Data Availability

The datasets generated and/or analyzed during the current study are not publicly available due to the fact that it is a database with more than 4 million records, which is too heavy, but are available from the corresponding author on reasonable request. Attached screenshot with the fields included in the raw data base.
